# FTY720 restores endothelial cell permeability induced by malaria sera

**DOI:** 10.1038/s41598-018-28536-1

**Published:** 2018-07-19

**Authors:** Karanyaporn Oggungwan, Supattra Glaharn, Sumate Ampawong, Srivicha Krudsood, Parnpen Viriyavejakul

**Affiliations:** 1 Department of Tropical Pathology, Faculty of Tropical Medicine, Mahidol University, 420/6 Rajvithi Road, Bangkok 10400, Thailand; 2 Department of Tropical Hygiene, Faculty of Tropical Medicine, Mahidol University, 420/6 Rajvithi Road, Bangkok 10400, Thailand

## Abstract

Increased endothelial cell (EC) permeability in severe *Plasmodium falciparum* malaria contributes to major complications of severe malaria. This study explored EC permeability in malaria, and evaluated the potential use of FTY720 to restore EC permeability. ECs were incubated with sera from malaria patients (*P*. *vivax*, uncomplicated and complicated *P*. *falciparum* malaria). Cellular permeability was investigated using a fluorescein isothiocyanate (FITC)-dextran permeability assay. FTY720, an analogue of sphingosine-1-phosphate (S1P), was tested for its potential action in maintaining EC integrity. ECs incubated with sera from malaria patients with complicated *P*. *falciparum* showed higher fluorescein leakage compared with ECs incubated with sera from *P*. *vivax* (*p* < 0.001) and uncomplicated *P*. *falciparum* (*p* < 0.001). ECs pretreated with FTY720 before incubation with malaria sera had significantly decreased fluorescein leakage compared with no FTY720 treatment. In addition, FTY720 treatment significantly reduced fluorescein leakage for both uncomplicated (at 45 min) (*p* = 0.015), and complicated *P*. *falciparum* malaria (15 min) (*p* = 0.043). The permeability increase induced by complicated *P*. *falciparum* sera was significantly reversed and prevented by FTY720 *in vitro*. FTY720 may have clinical applications to protect against endothelial barrier dysfunction in severe *P*. *falciparum* malaria.

## Introduction

Malaria is an important global health problem, especially in Africa and Asia. In 2016, the World Health Organization estimated there were 216 million cases of malaria with the highest incidence in the African region (90%)^1^. In Thailand, malaria periodically occurs in high risk areas, especially along the international borders of Thailand-Myanmar and Thailand-Cambodia. Severe complications are caused by the interaction between malaria parasites and the host, resulting in mechanical, immunologic, and humoral responses. The process of cytoadhesion between endothelial cells (ECs) and parasitised red blood cells (PRBCs) is an important factor in the pathogenesis of severe *Plasmodium falciparum* malaria. Cytoadhesion of PRBCs to the vascular ECs of different host organs along with rosette formation is considered the central mechanism of severe malaria^2^. Signalling events after cytoadhesion can cause injury to host tissues and trigger cellular changes such as apoptosis and cellular junctional changes^3,4^.

Sphingosine-1-phosphate (S1P) is a bioactive molecule that regulates cell growth, and suppresses apoptosis and survival^5^. S1P has an important role in controlling EC permeability by promoting cytoskeleton arrangement and restoring adherens junctions^6^. Previous studies on the use of S1P for the treatment of scalds and burns^7^ and acute lung injury/acute respiratory distress syndrome^8,9^ demonstrated S1P restored EC permeability. To date, no study has reported in detail on cell junctions between ECs in severe malaria. The present study explored the EC permeability in *P*. *falciparum* severe malaria. In addition, FTY720, an S1P analogue was evaluated for its potential use in protecting and restoring EC integrity and subsequently preventing fluid leakage caused by *P*. *falciparum* malaria.

## Results

### Cell permeability of endothelial cells induced by malaria sera

**Figure 1:**
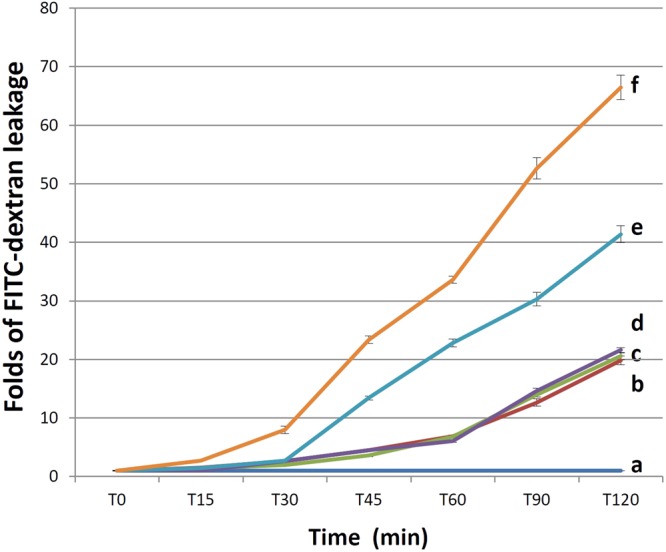
FITC-dextran leakage across the EC monolayer over time. ECs were incubated with media only (n = 12) (**a**), media + FITC-dextran (n = 12) (**b**), normal sera + FITC-dextran (n = 15) (**c**), sera from *P*. *vivax* + FITC-dextran (n = 15) (**d**), *P*. *falciparum* (uncomplicated) + FITC-dextran (n = 15) (**e**), or *P*. *falciparum* (complicated) + FITC-dextran (n = 16) (**f**).

This study investigated the changes in permeability of an EC monolayer exposed to malaria sera. Figure 1 shows the leakage of FITC-dextran through the EC monolayer over time. The starting fluorescence intensity was similar in all groups (all *p* > 0.05, media only = 243.08 ± 4.33, media + FITC-dextran = 237.17 ± 5.68, normal serum + FITC-dextran = 245.40 ± 7.96, *P*. *vivax* + FITC-dextran = 238.13 ± 9.64, *P*. *falciparum* (uncomplicated) + FITC-dextran = 248.20 ± 10.39, *P*. *falciparum* (complicated) + FITC-dextran = 246.25 ± 10.66). A flat fluorescence reading from T0–T120 was obtained from the media alone group, providing a good negative control for fluorescence recording (Fig. 1 line a). The media + FITC-dextran group showed a gradual leakage of fluorescein over time, which served as a baseline for FITC-dextran experiments (Fig. 1 line b). Similar fluorescence readings were observed in ECs induced with media + FITC-dextran, normal serum + FITC-dextran (Fig. 1 line c), and *P*. *vivax* serum + FITC-dextran groups (Fig. 1 line d) (all *p* > 0.05). ECs induced by uncomplicated or complicated *P*. *falciparum* showed significant FITC-dextran leakage (Fig. 1 line e, f, respectively). Permeability changes induced by sera from complicated *P*. *falciparum* was noted at 15 min (all *p* < 0.001), and at 45 min in uncomplicated *P*. *falciparum* (all *p* < 0.001). The highest FITC-dextran leakage was noted in ECs induced by complicated *P*. *falciparum* sera compared with media alone, normal serum, *P*. *vivax* serum, and *P*. *falciparum* uncomplicated groups (all *p* < 0.001, T15–T120). At the recording endpoint of T120, FITC-dextran leakage in ECs induced by complicated *P*. *falciparum* was increased more than 3-fold compared with normal sera and *P*. *vivax* sera, and was increased 1.6-fold compared with uncomplicated *P*. *falciparum*.

### Protective role of FTY720 in malaria sera induced endothelial cell permeability

**Figure 2:**
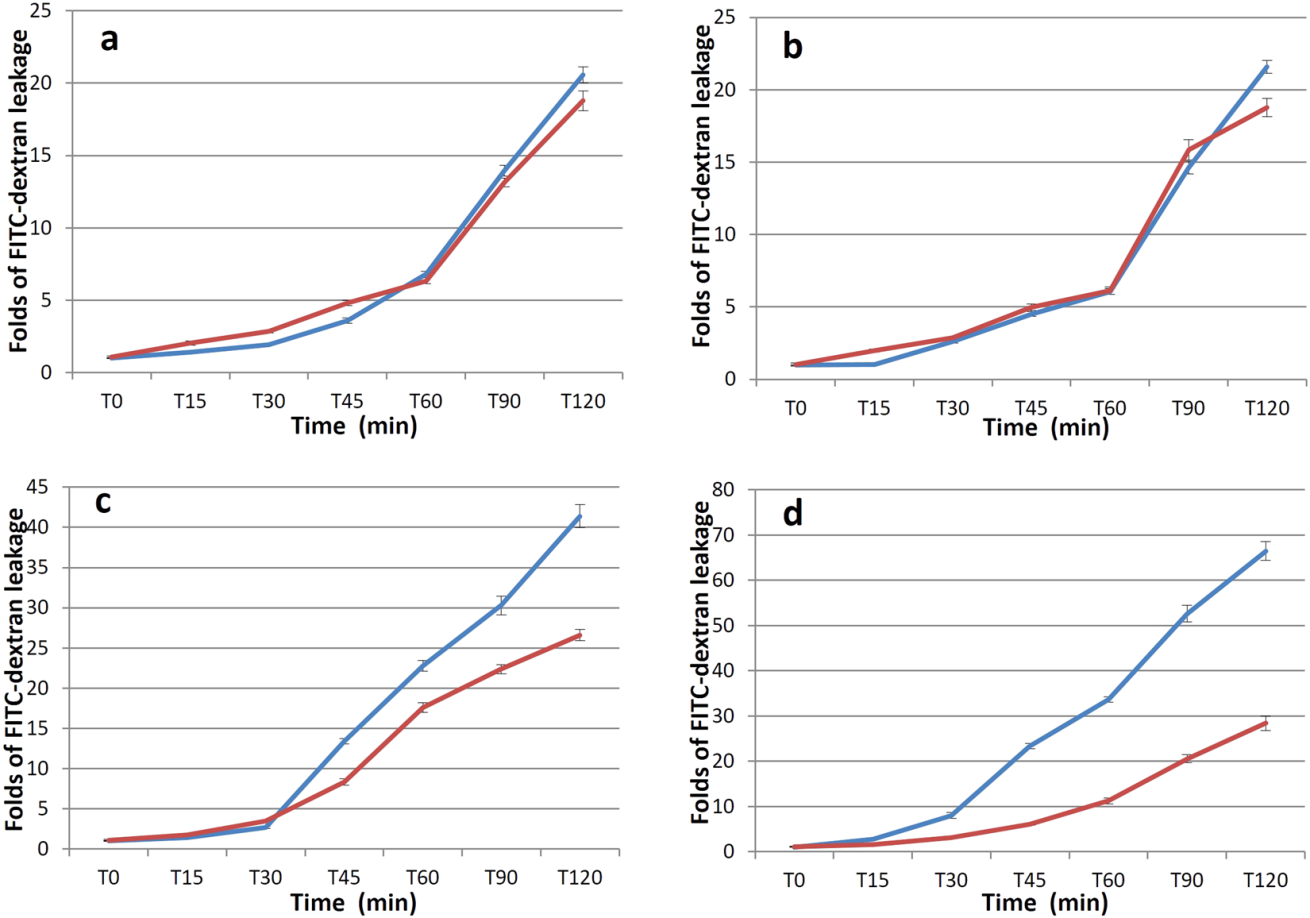
Comparison between FITC-dextran leakage of ECs pretreated with FTY720 (red line) and without FTY720 pretreatment (blue line) before incubation with normal sera (**a**), *P*. *vivax* sera (**b**), *P*. *falciparum* (uncomplicated) sera (**c**), or *P*. *falciparum* (complicated) sera (**d**). n = 11 per group.

To evaluate whether FTY720 protected barrier integrity, ECs were treated with FTY720 prior to incubation with sera from a normal volunteer, or a subject infected with *P*. *vivax*, uncomplicated *P*. *falciparum* or complicated *P*. *falciparum*. Results showed no difference in FITC-dextran leakage between FTY720 treatment and without FTY720 treatment in normal sera (*p* = 0.314, Fig. 2a) and *P*. *vivax* sera (*p* = 0.396, Fig. 2b) groups. For *P*. *falciparum* groups, a decrease in FITC-dextran leakage was observed in FTY720 treated groups. Differences in FITC-dextran leakage were noted at 45 min in uncomplicated malaria (*p* = 0.018, Fig. 2c), and at 30 min in complicated malaria (*p* = 0.043, Fig. 2d).

### Ability of FTY720 to reverse endothelial cell permeability induced by malaria sera

**Figure 3:**
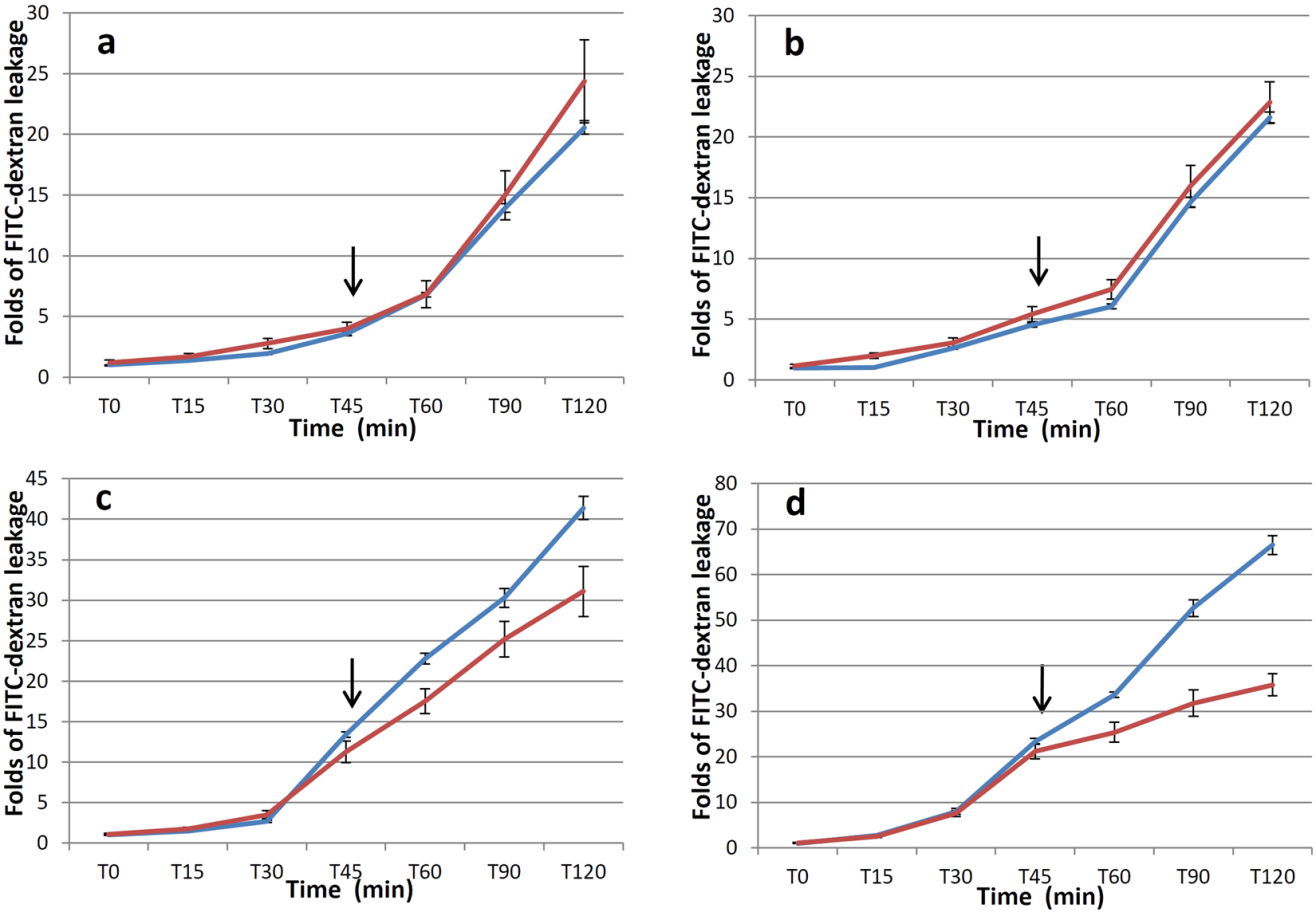
Comparison between FITC-dextran leakage of untreated ECs (blue line) and ECs treated with FTY720 (red line) after incubation with normal sera (**a**), *P*. *vivax* sera (**b**), *P*. *falciparum* (uncomplicated) sera (**c**), or *P*. *falciparum* (complicated) sera (**d**). The incubation period was from T0–T45. FTY720 was added to a final concentration of 1 µM at T45 (arrows). n = 11 per group.

ECs were incubated with sera from different experimental groups to increase their permeability, then FTY720 was used to treat the damaged ECs. Regardless of FTY720 treatment, FITC-dextran leakage was similar in normal sera (*p* = 0.331, Fig. 3a) and *P*. *vivax* (*p* = 0.108, Fig. 3b) groups. A significant decrease in FITC-dextran leakage was seen in the uncomplicated *P*. *falciparum* group after 45 min of FTY720 treatment, at T120 (*p* = 0.015, Fig. 3c), and in complicated *P*. *falciparum* after 15 min of FTY720 treatment, at T60 (*p* = 0.043, Fig. 3d).

## Discussion

EC dysfunction is a major factor in the development of vascular damage, an important consequence of complicated *P*. *falciparum* malaria. This study explored the effect of malaria sera on the cellular permeability of ECs and evaluated the function of FTY720 in protecting and reversing the cellular permeability. Using a co-culture system for EC-malaria sera, this study demonstrated that sera from *P*. *falciparum* malaria directly increased EC permeability *in vitro*, as measured by transwell permeability assays, with the highest FITC-dextran leakage in ECs exposed to sera from complicated *P*. *falciparum* malaria. The effect resulted in increased fluid leakage, which mimics clinical malaria complications such as pulmonary oedema and brain oedema. Endothelial leakage results from the loss of vascular integrity in response to various stimuli. Increased EC permeability has been reported in acute lung injury^10^, sepsis^11^, diabetes^12^, burn^13^, and infections such as severe dengue fever^14^ and malaria^15–18^. In malaria, an *in vitro* study on the effect of PRBCs on the integrity of human blood-brain barrier ECs showed a decrease in resistance, which was linked to the disruption of cell-to-cell junctions^16^. In addition, histidine-rich protein II, a malaria parasite virulence factor has been reported to cause a redistribution of endothelial junctional proteins and increase blood brain barrier permeability^17^. Similarly, ruptured PRBCs and the release of PRBC contents induced β-catenin activation, causing disruption of EC junctions in human brain microvascular ECs^18,19^. The integrity of the vascular EC layer is supported by tight junctions (TJ), adherens junctions (AJ), and gap junctions that are complexes located at the intercellular junction^20^. In malaria, alterations in junctional proteins, vascular endothelial (VE)-cadherin (a component of AJ)^21^, and zonula occludens (TJ protein)^4,22^ have been documented. Vascular barrier disruption inflicted on these junctional proteins in malaria infection causes sequential damage to cytoskeleton components including actin, intermediate filaments and microtubules.

In severe malaria, the process of cytoadhesion between PRBCs and ECs can cause direct damage to ECs resulting in the loss of barrier integrity. Chemical mediators dissolved in malaria sera are also important factors that enhance permeability changes. During malaria infection, soluble mediators such as tumour necrosis factor (TNF)-α, interleukin (IL)-6, 10^23^ and interferon (IFN)-γ^24^ are present in high concentrations and are associated with clinical severity, as well as adhesion receptors such as intercellular adhesion molecule (ICAM)-1, endothelial-leukocyte adhesion molecule (ELAM)-1 and E-selectin^25,26^. These mediators might contribute to the disruption of vascular integrity, cytoskeleton damage and subsequent increase in EC permeability.

An important focus of this study was the impact of FTY720 on EC permeability in malaria. As a treatment target, the study evaluated the benefit of FTY720 on barrier integrity by exposing ECs with malaria sera to induce an increase in vascular permeability, prior to treatment with FTY720. Here, the effect of FTY720 on permeability leakage was particularly evident in decreasing FITC-dextran leakage in ECs treated with severe *P*. *falciparum* sera. FTY720 had beneficial effects against damage induced by malaria sera on the EC barrier, in addition to its usefulness as a protective factor. In ECs of blood vessels, FTY720 maintained barrier integrity and prevented permeability leakage^27,28^. In experimental cerebral malaria, FTY720 prevented vascular leakage, inhibited neurological signs and prolonged animal survival^29,30^. Previous studies have shown that FTY720 induced the translocation of VE-cadherin to the contact sites at the EC junction^27,28^, and promoted cortical actin formation thereby stabilizing the AJ^31,32^. It would be interesting to assess the disruption of junctional molecules, which are complexed in TJ and AJ, i.e. occludin, claudins, junctional adhesion molecule (JAM), VE-cadherin, β-catenin, and the cytoskeleton changes in ECs exposed to malaria parasites/sera. In this study, the therapeutic efficacy and preventive effects of FTY720 to restore EC permeability exposed to malaria sera were demonstrated. A positive result with experimental FTY720 serves as a proof of concept to further explore the benefit of FTY720 as an adjuvant drug to prevent severe malaria complications such as cerebral oedema and pulmonary oedema.

## Materials and Methods

### Endothelial cell culture

Primary human umbilical vein endothelial cells (HUVECs) were purchased from the Japanese Collection of Research Bioresources Cell Bank (JCRB), Japan, and cultured according to the manufacturer’s instructions with modifications. One cryovial of primary HUVECs (1 ml) re-suspended in complete EC media (12 ml) (Gibco, Grand Island, NY, USA) was used to seed three gelatine-coated T25 cm^2^ vented cap flasks (Corning Inc., NY, USA) (4 ml per seeding vessel). The seeding vessels were coated with 2% bovine gelatine (Sigma-Aldrich, St. Louis, MO, USA) diluted to 1% gelatine in 1× phosphate buffered saline solution (PBS). HUVEC cultures were monitored under an inverted microscope daily. The EC media was changed the day after seeding and every other day thereafter. Cells were allowed to grow to >90% confluence (4–6 days), then HUVECs were subcultured. ECs used in the experiments were from passages 4–6. To grow ECs on the transwell filter, confluence adherent cells were trypsinised using trypsin-EDTA solution (1×) (Gibco, Grand Island, NY, USA), and seeded on the upper well of the Costar transwell filter (pore-size 0.4 μm) (Corning Inc., Pittston, PA, USA) coated with gelatine. Approximately 300,000–400,000 cells in 500 µl of cell suspension were loaded to the upper well. One cc of pre-warmed complete EC media was added to the lower well. Cells were maintained at 37 °C, 5% CO_2_ in a humidified incubator for 3–5 days or until >90% confluence.

### Blood Specimens

Stored sera from normal volunteers, *P*. *vivax* and *P*. *falciparum* malaria patients were used in this experiment. Controls were matched to patient sex and age. Malaria patients enrolled in the study were admitted at the Hospital for Tropical Diseases, Faculty of Tropical Medicine, Mahidol University, Thailand. Age range was from 16–58 years old. Sera used were from the day of admission before treatment. The clinical manifestations of severe malaria were based on WHO criteria^33^. Prior to storage, collected blood samples were left to stand at room temperature (RT) for 15–30 min to allow for clot formation. After centrifugation at 1,500 × g for 5 min, the sera were collected and stored at −80 °C until analysis. The study protocol was approved by the Ethics Committee, Faculty of Tropical Medicine, Mahidol University (MUTM 2014-055-01, with amendment, MUTM 2014-055-02 and MUTM 2014-055-03). All methods used during the investigation were performed in accordance with the relevant guidelines and regulations of the above institutional committee. The informed consent was obtained from all subjects.

### Cell permeability of endothelial cells induced by malaria sera

When ECs reached >90% confluence, complete EC media was removed from the upper well and replaced with 500 µl of new complete media containing malaria sera (10%) + Fluorescein isothiocyanate (FITC)-dextran conjugate (40 kDa; 1 mg/ml, Sigma-Aldrich, St. Louis, MO, USA)^34^. Media in the lower well was replaced by 1,000 µl fresh media only. To record the amount of fluorescein leakage, 100 µl of lysate was taken from the lower well immediately after incubation (Time (T) 0), then every 15 min until 2 hr (T15, T30, T45, T60, T90 and T120). One hundred µl of fresh complete media was added back thereafter. Special care was taken not to agitate the plate. The plate was constantly covered with aluminium foil to protect against the degradation of fluorescein by light. Lysate from the lower transwell was used to determine fluorescence at wavelengths of 485 nm and 528 nm^35^, using a Synergy H1 Hybrid Reader (BioTek, Winooski, VT, USA). Experimental groups consisted of *P*. *vivax* sera with FITC-dextran, uncomplicated *P*. *falciparum* sera with FITC-dextran and complicated *P*. *falciparum* sera with FITC-dextran (5 sera samples per groups with 2–4 replicates per group). Control groups included complete media alone (5 parallel experiments with 2–3 replicates per run), complete media with FITC-dextran (5 parallel experiments with 2–3 replicates per run), and normal sera with FITC-dextran (5 sera samples, each with 2–3 replicates).

### Protective role of FTY720 in malaria sera induced endothelial cell permeability

FTY720 (Sigma-Aldrich, St. Louis, MO, USA) at a final concentration of 1 µM^7^ was added to ECs on the upper transwell for 15 min. Then, FTY720 was removed and replaced with malaria sera (10%) containing FITC-dextran. The fluorescein leakage was determined as above at different time points.

### Ability of FTY720 to reverse endothelial cell permeability induced by malaria sera

ECs on the upper transwell filter were incubated with malaria sera for 45 min. Then, FTY720 containing FITC-dextran was added at a final concentration of 1 µM (at T45). The fluorescein leakage was assessed as above at different time points.

### Statistical analysis

Original data were expressed as the mean ± standard error of the mean (SEM). Fluorescence readings were quantified as ratiometric results relative to the control group. The normality of distribution was determined by the Kolmogorov-Smirnov test. Differences between groups were analysed by one-way ANOVA and the LSD multiple comparison test. Paired *t*-test was used to compare paired effectiveness of FTY720. A *p* value < 0.05 was considered statistically significant. Statistical analysis was performed using SPSS, version 18.0 (IBM, Ehningen, Germany).

### Availability of data and materials

All data generated or analysed during this study are included in this published article.
